# The effect of musical expertise on the representation of space

**DOI:** 10.3389/fnhum.2014.00250

**Published:** 2014-04-24

**Authors:** Carlotta Lega, Zaira Cattaneo, Lotfi B. Merabet, Tomaso Vecchi, Silvia Cucchi

**Affiliations:** ^1^Department of Psychology, University of Milano-BicoccaMilano, Italy; ^2^Brain Connectivity Center, National Neurological Institute C. MondinoPavia, Italy; ^3^The Laboratory for Visual Neuroplasticity, Department of Ophthalmology, Massachusetts Eye and Ear Infirmary, Harvard Medical SchoolBoston, MA, USA; ^4^Department of Brain and Behavioural Sciences, University of PaviaPavia, Italy

**Keywords:** musicians, pitch, space perception, line bisection, pseudoneglect

## Abstract

Consistent evidence suggests that pitch height may be represented in a spatial format, having both a vertical and a horizontal representation. The spatial representation of pitch height results into response compatibility effects for which high pitch tones are preferentially associated to up-right responses, and low pitch tones are preferentially associated to down-left responses (i.e., the Spatial-Musical Association of Response Codes (SMARC) effect), with the strength of these associations depending on individuals’ musical skills. In this study we investigated whether listening to tones of different pitch affects the representation of external space, as assessed in a visual and haptic line bisection paradigm, in musicians and non musicians. Low and high pitch tones affected the bisection performance in musicians differently, both when pitch was relevant and irrelevant for the task, and in both the visual and the haptic modality. No effect of pitch height was observed on the bisection performance of non musicians. Moreover, our data also show that musicians present a (supramodal) rightward bisection bias in both the visual and the haptic modality, extending previous findings limited to the visual modality, and consistent with the idea that intense practice with musical notation and bimanual instrument training affects hemispheric lateralization.

## Introduction

There is evidence that the pitch of a sound (i.e., the tone characteristic of being high or low and corresponding to its position in the musical scale, see Miller, 1916) may be represented in a spatial format referred to as the “mental pitch line” (see Rusconi et al., 2006). The earliest experimental evidence dates back to the last century when Pratt (1930) required individuals (with no musical training) to localize the position from which tones of different pitch seemed to come on a vertical scale (running from the floor to the ceiling). In that study, participants consistently positioned lower pitches in the lower section of the vertical scale, and higher pitches in the higher section of the vertical scale suggestive of a vertical mental representation of tones’ pitch (Pratt, 1930). More recently, converging findings (e.g., Rusconi et al., 2006; Lidji et al., 2007; Nishimura and Yokosawa, 2009; Cho et al., 2012) revealed that the mental pitch line affects manual motor responses: when an up or down response to a high or low pitched tone is required, individuals typically perform better for the mapping of the high tone to the up response (i.e., a key positioned in the upper part of the keyboard or response box) and the low tone to the down response (correspondingly, a key positioned in the lower part of the keyboard or response box) than for the opposite mapping. This preferential mapping has been found both when measuring accuracy (fewer errors for the up-high and low-down mapping than for the opposite one) and in terms of response latencies (faster responses for the up-high and low-down mapping than for the opposite one) (e.g., Rusconi et al., 2006; Lidji et al., 2007). Moreover, pitch height is also represented into an horizontal spatial dimension. Indeed, previous studies reported a preferential mapping for “high tones–right positions/low tones–left positions” than for “high tones–left positions/low tones–right positions” (e.g., Rusconi et al., 2006; Lidji et al., 2007). This effect has been labeled the *Spatial Pitch Association of Response Codes* (SPARC) (also the *Spatial-Musical Association of Response Codes, SMARC*) and it is modulated by both task features and by a participant’s level of musical expertise. For example, while the pitch of isolated tones has been found to automatically trigger the activation of a vertical axis independently of musical expertise (Rusconi et al., 2006; Lidji et al., 2007), the association of pitch along the horizontal axis seems to occur automatically only in musicians (at least, when response times are considered). Indeed, the SMARC effect in the horizontal plane may reflect spatial orthogonal stimulus-response compatibility mechanisms (see Rusconi et al., 2006; Nishimura and Yokosawa, 2009; see also Cho et al., 2012): a preferential up-right/down-left mapping has been consistently reported (e.g., Cho and Proctor, 2003), because a high pitch is spatially coded as “up” it would also be preferentially associated to the “right” (and vice versa for a low pitch). The association low-left and high-right in musicians does not seem to depend on the specific instrument played (e.g., Rusconi et al., 2006; Lidji et al., 2007) although it may be stronger in case of piano players, due to learned action–effect associations according to which left keys on the piano keyboard produce lower tones than right keys (see Stewart et al., 2004, 2013). In non musicians, this association low-left and high-right appears to occur only when participants have to process pitch intentionally (Rusconi et al., 2006; Lidji et al., 2007; Cho et al., 2012), but it has been reported also when pitch was irrelevant for the task if a reference tone was provided (Cho et al., 2012). Taken together, these results suggest that our cognitive system maps auditory pitch into a mental representation of space, that itself interacts with our motor responses, and that the association of pitch height with the vertical space seems to occur more automatically than the association of pitch height with the horizontal space (see also Vu et al., 2013).

The mental pitch line (Rusconi et al., 2006) is conceptually similar to the “mental number line” that characterizes the finding that number magnitude is also represented in a spatial format. Specifically, lower numbers tend to occupy leftward positions of the line, while higher numbers occupy rightward positions (Dehaene et al., 1993). Similarly, the mental number line has also been shown to affect motor responses. The SNARC (*Spatial Number Association of Response Codes*; Dehaene et al., 1993) effect refers to the observation that large numbers elicit faster responses with the right hand and small numbers elicit faster responses with the left hand.

The numerical magnitude and pitch of tones not only affect motor responses, but also how external space is represented. For instance, the presentation of numbers of different magnitude has been found to modulate spatial attention allocation as measured in Posner-like or line bisection paradigms (Fischer, 2001; Bonato et al., 2008; de Hevia and Spelke, 2009; Cattaneo et al., 2012a), with small numbers facilitating detection in the left hemifield and/or shifting line bisection judgments leftward, and large numbers facilitating detection in the right hemifield and/or shifting line bisection judgments rightward. Similarly, Ishihara et al. (2013) demonstrated that the simultaneous presentation of auditory pitches modulated performance in a visual line bisection task. In particular, listening to lower pitch tones shifted the bisection bias leftward, whereas listening to higher pitch tones shifted the bisection bias rightward in the horizontal plane (the effect of pitch height on vertical bisection being less clear) (Ishihara et al., 2013).

Beyond strengthening the association between pitch and manual motor responses (Rusconi et al., 2006; Lidji et al., 2007) musical training is also known to affect visuospatial attention which is likely to be represented more bilaterally in musicians than in non musicians (Patston et al., 2006, 2007a,b). For instance, when musicians perform the visual line bisection task, they do not show pseudoneglect (i.e., the tendency to bisect leftward to the real midpoint, see Jewell and McCourt, 2000), suggesting that musical experience may somehow influence the representation of peripersonal space and affect hemispheric lateralization (Patston et al., 2006). Indeed, pseudoneglect is believed to reflect a right hemispheric dominance in allocation of spatial attention (Foxe et al., 2003), resulting in an overrepresentation of the controlateral (left) side of space (Jewell and McCourt, 2000). Accordingly, a lesion to the right hemisphere (typically in parietal regions) may induce controlesional neglect, with patients’ attention being severely biased to the right side of space (e.g., Heilman and Van Den Abell, 1980). Musicians have been found to show a tendency to bisect to the right of the true midpoint (termed as “minineglect”, see Patston et al., 2006) and of a lower magnitude than the leftward bias shown by non musicians (this last finding suggests that intense musical training improves accuracy in bisecting visual lines). These findings suggest that spatial attention may be more balanced in musicians than in non musicians due to their extensive experience with reading music, itself a “spatial” language (i.e., a note identification exclusively depends on its spatial position on the staff), and intense bimanual instrument practice; both of which may overall enhance the development of spatial skills within the left hemisphere (Patston et al., 2006). Consistent with this view, when asked to indicate which hemifield a dot had been presented, musicians performed equally well with stimuli presented in both the left and right hemifield, whereas non musicians performed better for dots appearing in the left hemifield (Patston et al., 2007b). These findings further support the idea that early and extended musical training affects hemispheric representation of space (Patston et al., 2006, 2007b; see also Patston et al., 2007a, for neurophysiological evidence).

Up to now, the possible effects of pitch height on the perception and representation of external space have not been investigated in musicians compared to non musicians. Moreover, the effect of pitch on the representation of space has only been investigated in the visual modality (i.e., employing tasks requiring motor responses to visual stimuli) (e.g., Rusconi et al., 2006; Lidji et al., 2007; Nishimura and Yokosawa, 2009; Cho et al., 2012), or in a visual line bisection task (e.g., Ishihara et al., 2013). Whether pitch height also affects the representation of space in other sensory modalities (e.g., tactile/haptics) has not been previously investigated. In this study, we investigated the effects of pitch height on the representation of external space by using a crossmodal sensory paradigm. In a first experiment, we assessed whether musicians show a different bisection bias compared to non musicians in a haptic horizontal bisection task. Musicians skilled in different instruments were recruited for Experiment 1, in line with previous work assessing the same issue in the visual modality (Patston et al., 2006). Given converging evidence showing the occurrence of pseudoneglect in both the visual and haptic modalities (see Jewell and McCourt, 2000), we expected to find a rightward bias in musicians’ haptic bisection as previously reported for the visual modality (Patston et al., 2006). In a second experiment, we investigated whether pitch height differently affects the performance of musicians and non musicians in a line bisection task performed either visually or haptically (i.e., implying tactile exploration of the rods while wearing a blindfold). During the task, participants were presented with tones of high vs. low pitch, that could be either relevant or irrelevant for a judgment participants had to make following bisection (i.e., pitch vs. timbre of a note). Only piano players were included in Experiment 2 (as in Stewart et al., 2013): although the occurrence of a SMARC effect does not depend on the specific instrument played in musicians (Rusconi et al., 2006; Lidji et al., 2007; Cho et al., 2012), we preferred to have a more homogeneous group, also in light of previous evidence suggesting that the SMARC effect may be stronger in pianists due to learned action–effect associations (Lidji et al., 2007; see also Stewart et al., 2004, 2013). We expected pitch height to modulate musicians’ bisection performance in both conditions when pitch height was relevant and when it was irrelevant for the task, in light of previous evidence suggesting that this cue is automatically represented by musicians in a horizontal spatial dimension (Rusconi et al., 2006; Lidji et al., 2007; Cho et al., 2012). We expected pitch height to also consistently modulate non musicians’ performance when pitch had to be intentionally processed (Rusconi et al., 2006; Lidji et al., 2007). In turn, we expected pitch to have no (Rusconi et al., 2006; Lidji et al., 2007) or at most, a weak effect (Ishihara et al., 2013) on non musicians’ bisection performance when pitch was task irrelevant. Finally, we expected to find comparable cueing effects in both the haptic and visual modalities, in light of previous evidence reporting similar effects of numerical cues on visual and haptic bisection (Cattaneo et al., 2012a).

## Methods

### Participants

In Experiment 1, twelve right-handed (Oldfield, 1971) musicians with no history of neurological conditions took part in the experiment (7 males; mean age = 24.33 years, *SD* = 4.69, range 19–35; mean years of instrument experience = 11.17, *SD* = 2.86, range 7–16). Four were guitarists, two clarinettists, two flutists and four pianists and all had passed at least the fifth exam of instrument proficiency in an Italian Conservatory of Music (the fifth exam corresponds to a high level of proficiency and implies intensive study of music for an average of 7 years).

In Experiment 2, 12 skilled piano players (4 males, mean age = 24.4 years, *SD* = 4.33, range 20–36; mean years of piano experience = 14.5, *SD* = 2.94, range 10–20) and 12 non musicians (4 males, mean age = 23.25 years, *SD* = 1.42, range 21–26) took part in the experiment. All participants of Experiment 2 were right-handed (Oldfield, 1971) and had normal hand function and no auditory problems. As for Experiment 1, all musicians had passed at least the fifth exam of instrument proficiency in an Italian Conservatory of Music. The inclusion criterion for non musicians was to have no musical experience beyond a basic level acquired during middle school (typically implicating basic practice with a recorder instrument). None of the musicians taking part in Experiment 2 had participated in Experiment 1.

### Stimuli

In Experiment 1, stimuli consisted of wooden rods of five different lengths (30, 35, 40, 45, 50 cm) all with a diameter of 14 mm. The rods were positioned horizontally, with respect to the midline and presented on a table at a fixed distance of 38 cm. Each rod was fixed with Velcro strips horizontally onto a wooden panel. The rods could thus be haptically explored without being moved and a constant alignment between the participant’s mid-sternum and the midpoint of the rod could be maintained (see Baek et al., 2002; Cattaneo et al., 2011).

In Experiment 2, the rods used were the same as those used in Experiment 1. In this task, auditory stimuli were presented and varied according to the experimental condition (see Section Procedure). In the “height judgment” condition, auditory stimuli consisted of two pure low tones (C3, 131 Hz and G3, 196 Hz) and two pure high tones (E5, 659 Hz and B5, 988 Hz) (for further details see Lidji et al., 2007). In the timbre judgment condition, auditory stimuli consisted of two pure tones (C3 and B5), and two distorted tones (C3 and B5 distorted; i.e., in which the physical shape of the original tone was changed leaving pitch height unaffected). In the control condition, participants were presented with white noise in order to provide a baseline measure for neutral auditory stimulation (see also Ishihara et al., 2013). All the auditory stimuli were created using the software Audacity and reproduced with Quick Time Player using a MacBook computer.

### Procedure

In Experiment 1, participants were blindfolded throughout the entire experiment. They were instructed to explore the length of the rod in their preferred direction (left-to-right or right-to-left) using their index finger only. At the beginning of each trial, the experimenter placed the palm of the participant’s hand on the rod, such that it covered approximately the midpoint of the rod (i.e., the center of the palm was a few millimetres off from the true midpoint). This palm-based starting position could not be used as an accurate estimate of the line’s midpoint, due to its approximate nature and because at the start of each trial, participants were instructed to lift their palm off the rod and begin exploring it with their index finger. This starting point for haptic exploration was used in order to control for systematic biases in scanning direction that may have influenced bisection performance (see Baek et al., 2002; see Cattaneo et al., 2011, 2012a, b, for a similar procedure). We did not place the finger of the participants directly on the midline to avoid influence based on memory of the original position to be used as a “reference” for the bisection. On each trial, the participants were given 6 s to scan the rod, and they could do so as many times as they wished. At the end of each trial, they were asked to indicate the midpoint of the rod by positioning their index finger over it. At the start of the experiment, a vertical line (approximately 1 mm wide) was drawn in the middle of the tip of participants’ index fingers. After each trial, the experimenter used this line to measure (using a measuring tape) the difference between the participants’ perceived midpoint and the actual midpoint, to the nearest millimetre. No feedback was given to the participants during the task. The experimental block consisted in the presentation of 15 rods (each of the five rods was presented three times), and was performed once with the left hand and once with the right hand (order of hand execution was counterbalanced across participants). Rods of different length were presented in a random order but the same rod was never presented consecutively. The experiment started with a practice session (results not included in the analyses), in which participants were instructed to bisect two rods with the left hand, and two rods with the right hand (the rod length used in the practice varied across participants).

In Experiment 2, the task required participants to carry out a haptic bisecting task (using the same rods of the previous experiment) while listening to different auditory stimuli. The same task was presented: (1) in two modalities: haptic and visual and (2) under two task conditions: a pitch judgment task (requiring to focus attention to the pitch of the tones) and a timbre judgment task (for which pitch height was irrelevant). The bisection task was performed with the right hand only and in the haptic condition, participants were blindfolded throughout the entire experiment.

In the visual condition, participants were instructed to look at the rods and indicate the midpoint of the bar using their right index finger. A maximum response time of 6 s was allowed. During error measurement, the measuring tape was positioned in such a way that numbers were visible only to the experimenter. In the haptic condition (as for Experiment 1), participants were instructed to explore the length of the rod (either left-to-right or right-to-left) and as many times as they wished, during a 6 s limit.

During the tactile and visual exploration, participants were simultaneously presented (via headphones) with an auditory stimulus lasting 6 s. In the visual trials, the auditory stimulus started 3 s before the start of the bisection task. A wooden panel was placed in front of the to-be-explored rod to prevent participants from seeing it, and it was then removed 3 s after the sound was presented. This ensured that participants had processed the sounds before performing the bisection task (see Cattaneo et al., 2012a, for a similar procedure). In the haptic trials, the stimulus started with the beginning of the exploration. Six seconds were given for haptic exploration and the sound duration covered the entire exploration period. Previous findings have shown that auditory cues presented concurrently with the tactile exploration were effective in modulating the bisection bias (Cattaneo et al., 2012a, b). Participants were instructed to pay attention to the sounds. Following bisection, in the height judgment task, participants had to verbally indicate whether the auditory stimulus was a low tone, a high tone, or consisted of white noise. In the timbre judgment task, participants had to verbally indicate whether the auditory stimulus was a normal tone, a distorted tone, or consisted of white noise. In each condition, the auditory stimuli, as well as different lengths of rods, were presented in random order. In each task (height and timbre judgment) and for each modality (visual and haptic), each of the five rods was presented five times (once for each different sound). Hence, there were 50 trials in the visual modality (25 for the height and 25 for the timbre task), and 50 trials in the haptic modality (25 for the height and 25 for the timbre task). Trials were presented in blocks for task condition (height vs. timbre) and modality (visual vs. haptic). The order of task and modality was counterbalanced across participants. The entire experiment lasted approximately 2 h (included breaks between conditions).

Before performing the real experiment, participants were presented with a practice session (results not included in the analyses), in which they were instructed to bisect each of the five lengths rods within the 6 s limit both in the visual and in the haptic modality. They were also taught which tones were identified as low and high, and the distinction between a pure and distorted tone. No feedback on performance was given to the participants during the testing.

### Data analysis

#### Experiment 1

For the data analysis, deviations from the veridical center were converted into signed percentage scores (positive if bisections were to the right and negative if bisections were to the left) by subtracting the true half-length of the rod from the measured distance of each setting from the left extremity of the rod and then dividing this value by the true half-length and multiplying the quotient by 100 (see Laeng et al., 1996; Cattaneo et al., 2011). Signed percentage deviations for the five different lengths were collapsed together in the following analyses. In addition, the standard deviations of the bias scores were calculated and analyzed separately as measures of variability of judgments (variable error, see Laeng et al., 1996; Martinez-Cascales et al., 2013).

#### Experiment 2

The signed percentage bisection bias was computed as in Experiment 1. One sample *t*-tests were used to compare the average bisection bias in the control (white noise) conditions against zero (i.e., the true midline, absence of bisection directional bias), collapsing across the two tasks (height and timbre). A mixed repeated measures ANOVA, with condition (low tone, high tone, white noise) as within-subjects variables and group (musicians vs. non musicians) as a between-subjects variable was performed on the signed percentage scores and on the variable error (i.e., standard deviations) reported in each task (pitch height vs. timbre) separately. Bonferroni correction was applied to all *post-hoc* comparisons.

Accuracy in the height and timbre judgments was at ceiling in both groups of participants in both the visual and the haptic conditions (mean accuracy >98% in all conditions), and was not further considered in the analysis. The high performance in the auditory tasks confirmed that participants did indeed pay attention to the sounds.

## Results

### Experiment 1

**Figure 1:**
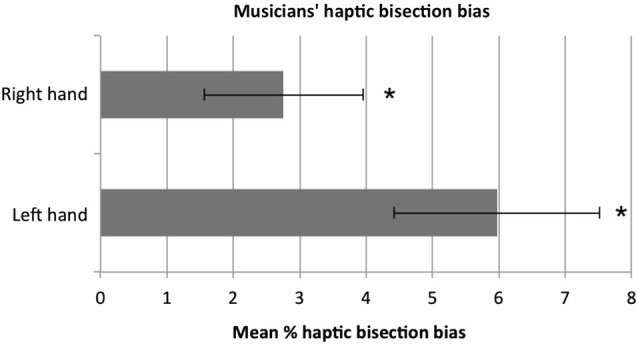
**The mean percentage bisection bias in musicians**. Participants showed a significant rightward bias (minineglect) both with the left and with the right hand. The tendency to bisect to the right was stronger with the left than with the right hand, although not to a significant extent (*p* = 0.081). Error bars represent ±1 SEM. Asterisks indicate that the bias was significantly different from zero (i.e., true midline, absence of bias).

The mean bisection percentage bias for the left and the right hand is shown in Figure 1. A one-sample *t*-test against zero (i.e., no bias) showed that musicians on average significantly bisected the rod to the right of the true midpoint with both the right, *t*_(11)_ = 2.31, *p* = 0.041, and the left hand, *t*_(11)_ = 3.86, *p* = 0.003. Moreover, the rightward bias tended to be larger when the left hand was used compared to the right, however the difference in performance between the two hands did not reach significance (*p* = 0.081). Although the low numbers of musicians for each instrument category does not allow us to make any reliable parametric comparisons, a qualitative inspection of the data showed that musicians tended to bisect rightward regardless of the instrument played. When participants’ variable error (i.e., standard deviations) was considered, no difference between the two hands was reported, *t*_(11)_* =* 1,* p =* 0.410. Correlational analyses (Pearson, two-tailed) were performed to assess whether level of musical expertise correlated with the bisection bias. No significant correlation was found for either the right (*r =* 0.438, *p =* 0.157) or the left (*r =* 266, *p =* 0.403) hand.

### Experiment 2

#### Visual bisection

**Figure 2:**
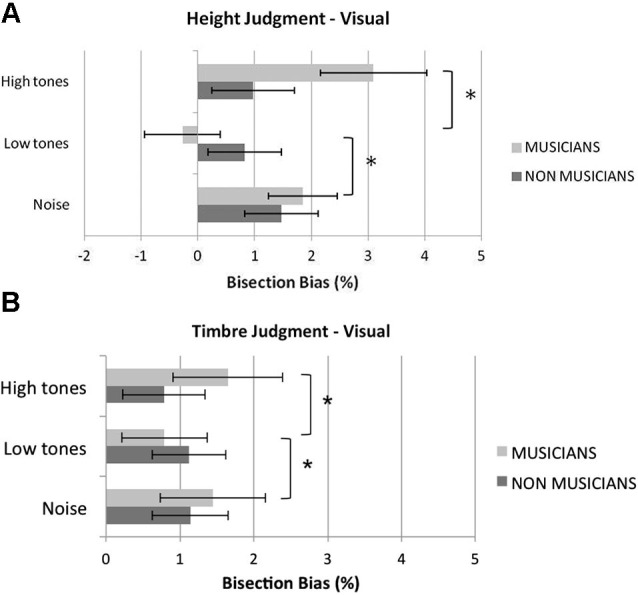
**Mean percentage visual bisection bias in the height (A) and in the timbre (B) judgment tasks in visually bisecting rods in the control (white noise), low tones and high tones conditions**. Overall, listening to low tones shifted musicians’ bisection significantly to the left compared to listening to white noise or high tones; pitch of the tones did not affect non musicians’ performance. Error bars represent ±1 SEM. Asterisks indicate significant differences between task conditions.

Figure 2 shows musicians and non musicians’ mean bisection bias in the different experimental conditions of the visual bisection task. In the baseline white-noise condition, a significant rightward bias was reported in both musicians, *t*_(11)_ = 2.86, *p* = 0.016, and non musicians, *t*_(11)_ = 2.95, *p* = 0.013. The overall mean variable error (i.e., standard deviations of the bias scores) was comparable in musicians and non musicians, *t*_(22)_ < 1, *p =* 0.416, suggesting comparable precision in the two groups.

In the *height judgment task* (Figure 2A), the analysis revealed a significant main effect of condition, *F*_(2,44)_ = 10.00, *p* < 0.001, $\eta_{p}^{2}=0.31$, and a significant interaction condition by group, *F*_(2,44)_ = 7.65,* p* = 0.001, $\eta_{p}^{2}=0.26$. The main effect of group was not significant,* F*_(1,22)_ = 0.28, *p* = 0.61, $\eta_{p}^{2}=0.012$. The main effect of condition was further analyzed in light of the significant interaction condition by group. An analysis of the simple effect of condition within each group showed that condition was not significant for non musicians, *F*_(2,22)_ = 2.49,* p* = 0.11, $\eta_{p}^{2}=0.19$, whereas it was significant in the musician group, *F*_(2,22)_ = 9.79, *p* = 0.001, $\eta_{p}^{2}=0.47$. Pairwise comparisons revealed that in musicians the low tones shifted the perceived midline significantly to the left compared to the white noise condition, *t*_(11)_ = 4.28, *p* = 0.004, and to the high tones condition, *t*_(11)_ = 3.32, *p* = 0.021. Conversely, the bisection bias shown in the high tone condition was not significantly different from that shown in the white noise condition, *t*_(11)_ = 1.77, *p* = 0.31.

In the *timbre judgment task* (Figure 2B), the main effect of condition was not significant, *F*_(2,44)_ = 1.14, *p* = 0.33, $\eta_{p}^{2}=0.049$; however, the interaction condition by group reached significance, *F*_(2,44)_ = 3.35, *p* = 0.044, $\eta_{p}^{2}=0.13$. The main effect of group was not significant, *F*_(1,22)_ = 0.12, *p* = 0.74, $\eta_{p}^{2}=0.005$. An analysis of the simple effect of condition within each group showed that condition was not significant for non musicians, *F*_(2,22)_ = 0.50,* p* = 0.61, $\eta_{p}^{2}=0.044$, whereas it was significant in the musician group, *F*_(2,22)_ = 7.36, *p* = 0.004, $\eta_{p}^{2}=0.40$. Pairwise comparisons revealed that in musicians, the low tones shifted the perceived midline significantly to the left compared to both the white noise condition,* t*_(11)_ = 3.14, *p* = 0.028, and the high tones condition, *t*_(11)_ = 3.19, *p* = 0.026, whereas no differences in the bisection bias were reported between the white noise condition and the high tone condition, *t*_(11)_ < 1, *p* = 0.37.

#### Haptic bisection

**Figure 3:**
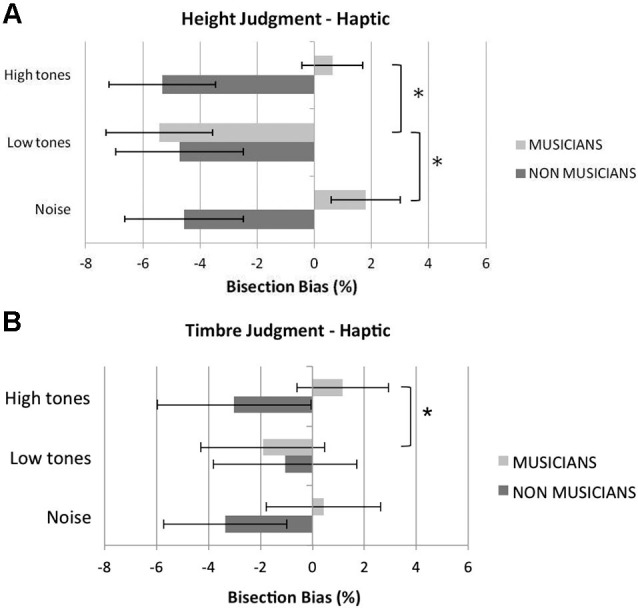
**Mean percentage haptic bisection bias in the height (A) and in the timbre (B) judgment tasks in bisecting rods in the control (white noise), low tones and high tones conditions**. Overall, listening to low tones shifted musicians’ bisection significantly to the left compared to listening to white noise or high tones. Pitch of the tones did not affect non musicians’ performance. Error bars represent ±1 SEM. Asterisks indicate significant differences between task conditions.

Figure 3 shows musicians and non musicians’ mean bisection bias in the different experimental conditions of the haptic bisection task. Overall, in the baseline white-noise condition, musicians showed a tendency to bisect to the right of the veridical midpoint, but this deviation was not significant, *t*_(11)_ < 1, *p =* 0.45. Non musicians significantly bisected to the left of the veridical midpoint, *t*_(11)_ = 2.30, *p =* 0.042. Musicians and non musicians did not differ in their overall variable error, *t*_(22)_ < 1,* p* = 0.441, suggesting comparable precision in the two groups (as in the visual task). Moreover, musicians and non musicians did not significantly differ in their haptic exploration strategy (i.e., number of scanning movements), *t*_(22)_ < 1,* p =* 0.860 (mean number of explorations for musicians = 4.17; for non musicians = 4.25), thus ruling out a possible role of this factor in contributing to differences observed in the bisection bias.

In the *height judgment task* (Figure 3A), the analysis revealed a significant main effect of condition, *F*_(2,44)_ = 9.00, *p* = 0.001, $\eta_{p}^{2}=0.29$, and a significant interaction condition by group, *F*_(2,44)_ = 9.64, *p* < 0.001, $\eta_{p}^{2}=0.31$. The main effect of group was not significant, *F*_(1,22)_ = 2.89, *p* = 0.10, $\eta_{p}^{2}=0.12$. The main effect of condition was further analyzed in light of the significant interaction condition by group. An analysis of the simple effect of condition within each group showed that condition was not significant for non musicians, *F*_(2,22)_ < 1, *p* = 0.70, $\eta_{p}^{2}=0.03$, whereas it was significant in the musician group, *F*_(2,22)_ = 12.48, *p* < 0.001, $\eta_{p}^{2}=0.53$. Pairwise comparisons revealed that in musicians the low tones shifted the perceived midline significantly to the left compared to the white noise condition, *t*_(11)_ = 6.74, *p* < 0.001, and to the high tones condition, *t*_(11)_ = 3.21, *p* = 0.025. Conversely, the bisection bias shown in the high tone condition was not significantly different from that shown in the white noise condition, *t*_(11)_ < 1, *p* = 0.48.

A similar ANOVA performed for the *timbre judgment task* (Figure 3B) revealed no significant main effect of condition, *F*_(2,44)_ = 0.31, *p* = 0.74, $\eta_{p}^{2}=0.01$; however, the interaction condition by group reached significance, *F*_(2,44)_ = 5.96, *p* = 0.005, $\eta_{p}^{2}=0.21$. The main effect of group was not significant, *F*_(1,22)_ < 1, *p* = 0.48, $\eta_{p}^{2}=0.02$. An analysis of the simple effect of condition within each group showed that condition was not significant for non musicians, *F*_(2,22)_ = 2.18, *p* = 0.14, $\eta_{p}^{2}=0.17$, whereas it was significant in the musician group,* F*_(2,22)_ = 4.25, *p* = 0.028, $\eta_{p}^{2}=0.28$. Pairwise comparisons revealed that in musicians the low tones shifted the perceived midline significantly to the left compared to the high tones condition, *t*_(11)_ = 3.67,* p* = 0.012, whereas no differences in the bisection bias were reported between the white noise condition and either the low tone, *t*_(11)_ = 2.44, *p* = 0.29, or the high tone condition, *t*_(11)_ < 1, *p* = 0.53.

Possible differences in the initial scanning direction induced by the auditory stimuli were also analyzed. Percentage of trials in which exploration started to the left vs. to the right was computed. One-sample *t*-tests against 50% (i.e., no preferential initial scanning direction) were carried out to verify whether in the white-noise baseline condition (collapsed for timbre and height task) musicians and non musicians showed a preferential initial scanning direction. Non musicians showed a tendency to start exploring the rod to the left (this was the case in 70% of the trials), but it did not reach full significance, *t*_(11)_ = 1.72, *p* = 0.11. In turn, musicians showed an opposite tendency starting exploration preferentially to the right (this was the case in 66.7% of the trials), but again this tendency failed to reach full significance, *t*_(11)_ = 1.82, *p* = 0.096. In particular, seven non musicians and four musicians always started exploration towards the same side of the rod. A pairwise comparison performed on the other participants who varied their initial scanning direction across trials, revealed no difference in the directional bias depending on the initial scanning direction, *t*_(12)_ < 1, *p* = 0.983. Hence, a repeated measures ANOVA with condition (low pitch, high pitch, white-noise) as a within-subjects variable and group as a between-subjects variable was performed on the starting scanning directions values for each task (height and timbre). *In the pitch judgment task*, the analysis revealed a significant main effect of group, *F*_(1,22)_ = 9.30,* p* = 0.006, reflecting musicians tendency to starting exploration to the right, and non musicians tendency to starting exploration to the left. Neither the main effect of condition (*p* = 0.77) nor the group by condition interaction (*p =* 0.47) were significant, indicating that the different auditory stimuli did not significantly affect the initial scanning direction. In the timbre judgment task the analysis revealed an almost significant main effect of group *F*_(1,22)_ = 3.99, *p* = 0.058, reflecting the opposite tendency in the starting direction found in the baseline noise condition. The main effect of condition (*p* = 0.40) and the interaction group by condition (*p* = 0.93) were not significant.

## Discussion

The results of Experiment 1 demonstrate that musicians show a consistent tendency to bisect to the right of the veridical midpoint in a haptic bisection paradigm. This finding corroborates and extends previous evidence reported in the visual modality (Patston et al., 2006). The results of Experiment 2 demonstrate that pitch height influences the representation of visual and haptic horizontal space (measured via a bisection paradigm) in musicians, but not in non musicians. This was the case both when pitch height was relevant for the task, and when it was irrelevant (although in latter case, the effect of pitch on the bisection bias was weaker in the haptic modality). Notably, the effect of pitch height on space representation was limited to low-tones that induced a significant leftward shift in the bisection bias of musicians, whereas listening to high-pitch tones did not affect the bisection bias differently than listening to a neutral auditory condition (white-noise).

Previous studies comparing behavioral performance of musicians and non musicians have revealed an influence of pitch on motor responses, as an indexing the association between low tones and left responses and high tone and right responses (Rusconi et al., 2006; Lidji et al., 2007; Nishimura and Yokosawa, 2009; Cho et al., 2012; see also Vu et al., 2013). However in non musicians, this effect was reported only when pitch height had to be attended to intentionally (but see Cho et al., 2012), whereas in musicians, the association occurred even when pitch height was irrelevant for the task. Our findings in musicians appear in line with this previous evidence (Rusconi et al., 2006; Lidji et al., 2007; Cho et al., 2012) suggesting that in musicians, tones are likely to automatically activate a “music spatial line” that is oriented left to right. Indeed, pitch height is likely to be treated by the brain as an ordinal sequence, as with the order of days of the week, months of the year, or the alphabet; with ordinal sequences being mentally represented in a left-to-right direction (Gevers et al., 2003). In the case of musicians who are piano players, this may be even more strongly evident due to low notes being produced by left keys and high notes being produced by right keys on the piano keyboard (see Stewart et al., 2004, 2013). Our data show that the activation of such spatial representation of tone height in musicians (or at least in piano players such as in our participants) is able to interfere with the representation of external space, visually or haptically perceived, resembling previous evidence reported in the numbers’ domain (see Cattaneo et al., 2012a). The modulation of pitch height on the bisection bias was observed in musicians both when pitch height had to be attended to, and when it was irrelevant (timbre judgment condition), although in the latter condition effects were less pronounced in the haptic modality. These findings suggest that pitch height automatically activates a “music mental line” in musicians (although the effect may be stronger when pitch is intentionally processed), supporting previous evidence (Rusconi et al., 2006; Lidji et al., 2007), and again showing strong resemblance with the effects exerted by numbers on spatial representation (e.g., Dehaene et al., 1993; Fias et al., 1996; Bonato et al., 2008; Cattaneo et al., 2012a).

Critically in musicians, the effect of pitch tones was reported only for low pitches that shifted the bisection bias significantly leftward, whereas listening to high tones did not significantly shift the pre-existing rightward bias further to the right. White noise has been previously used as a control auditory stimulus (e.g., Ishihara et al., 2013; Mendonça et al., 2013). Although all frequencies are equally represented in white noise, the sound is perceived as higher-pitched to human observers, partly because the perception of pitch is not linear, and partly because human ears are more sensitive to higher frequencies (Plack, 2005). This may partly explain why in our experiment the high-tones did not produce a significant modulation of the response bias compared to the baseline (white-noise) condition. Moreover, it is possible that only low pitch tones influenced bisection errors in musicians because they have a tonic rightward bisection error which is “counteracted” (i.e., moved leftward) by the low pitch tones. A similar argument has been made with respect to the influence of left and right visual cues on visual line bisection (McCourt et al., 2005), where the effect of cues delivered to the end of lines was more effective if they counteracted an existing bias (see also Tamietto et al., 2005; Cattaneo et al., 2012a, 2013). Indeed, when a cue is added to the pre-existing bias in bisection (as in our experiment high pitch tones that are likely to occupy the right portion of the putative music line in musicians), a threshold point may be reached at which errors are no further tolerated by the perceptual system and corrections are taken (McCourt et al., 2005; Laeng et al., 1996).

There was no evidence for the influence of sound pitch over spatial representation in the non musician group we tested. This finding may appear in contrast to previous findings (Rusconi et al., 2006; Lidji et al., 2007; Nishimura and Yokosawa, 2009; Cho et al., 2012) who reported that there is an association between pitch height and the horizontal space when pitch had to be attended intentionally. However, in these previous studies this pattern was evident only when response latencies were considered, with no effect on participants’ accuracy (with the exception of Cho et al., 2012, that also reported an effect on accuracy in one of their experiments). Conversely, an association between the vertical space and pitch height was more consistently observed across different measures (i.e., accuracy and reaction times, the latter being affected even when the pitch height was irrelevant for the task) (see Rusconi et al., 2006) suggesting that a left-right remapping of the low-high height dimension of tones is weaker than a more direct remapping of tone height into a down-up direction in non musicians. Moreover, in this study, we used a line bisection paradigm, which represents a direct estimate of the external space while previous studies (Rusconi et al., 2006; Lidji et al., 2007; Nishimura and Yokosawa, 2009; Cho et al., 2012) measured response compatibility effects that may be more vulnerable to the influence of a simultaneously activated spatial representation. Indeed, in a line bisection task, the effect of a concurrently activated spatial mental representation needs to overcome a physically perceived space (that may be more “robust” to interference), whereas this is not the case when a fast motor response in space is required. Finally, in a recent study using visual line bisection in non musicians, Ishihara et al. (2013) reported that the concurrent presentation of tones of different pitches (high or low) modulated performance with low tones shifting the bisection bias leftward, and high tones shifting the bisection bias rightward. However, the effect of tones pitch on the vertical bisection bias was weaker than in case of horizontal bisection (Ishihara et al., 2013, Experiment 1) in contrast with prior evidence suggesting that the SPARC effect is stronger in the vertical plane (Rusconi et al., 2006; Lidji et al., 2007; see also Vu et al., 2013). Moreover, the modulatory effect of pitch on bisection was found only when low and high tones were precisely intermixed in a repeated order (i.e., low, high, low, high,…) and not when they were presented in blocks. The fact that the tone presentation was not randomized (as it was in our study) in the alternate presentation, together with the fact that only one line length was used and that participants’ music experience was not controlled (as stated by the authors Ishihara et al., 2013, see footnote 2) may have somehow affected task sensitivity and overall performance. Finally, a baseline auditory condition was not included in that paradigm (Ishihara et al., 2013, Experiment 1) so it is not clear whether the effects were driven by the low or by the high pitch. Overall, these differences in the paradigm may account for the discrepancy of our results with those by Ishihara et al. (2013).

In the baseline (white-noise) condition of Experiment 2, non musicians showed a rightward deviation in the visual modality and a leftward deviation in the haptic modality. The rightward deviation may appear surprising since individuals who are non musicians typically bisect to the left of the veridical midpoint. However, listening to white-noise *per se* is known to induce a shift in the bisection bias compared to a silent condition due to alertness effects affecting hemispheric imbalance (Cattaneo et al., 2012b). The white-noise may have simply reduced the leftward bias in the haptic modality (as in Cattaneo et al., 2012b) and even reversed it to an opposite bias in the visual modality. This cannot be directly assessed in our data, since we did not include a silent baseline condition in Experiment 2 given that our goal was to have a baseline “auditory” condition to control for unspecific effect of auditory stimulation.

Finally, the findings of Experiment 1 show that musicians (professional in playing piano or other instruments) tend to bisect rightward in haptic line bisection, confirming and extending to the haptic domain evidence so far only available for the visual modality (Patston et al., 2006). These data show that the rightward bias in musicians is “supramodal”, in line with previous evidence showing that pseudoneglect (i.e., a leftward spatial bias) also occurs across different sensory modalities (see Jewell and McCourt, 2000). According to Patston et al. (2007b), spatial attention in musicians is likely to be more bilaterally controlled than in non musicians, in which the right hemisphere is usually dominant in spatial tasks, possibly due to long-term practice of musical reading, and intense bimanual exercise. Our data also suggest that the tendency to deviate rightward did not depend on playing a specific instrument, an aspect that was not considered by Patston et al. (2006). Patston et al. (2006) also suggested that musicians are more accurate than non musicians in line bisection. However, we did not find evidence for an higher accuracy of musicians compared to non musicians in either visual or haptic bisection when the variable error (i.e., mean of the standard deviations of the bias scores representing a measure of variability of judgments) was considered (see Section Experiment 2). Moreover, it is worth noting that we observed a trend (not significant) toward a larger rightward deviation with the left than with the right hand, an effect that did not depend on overall precision, since both hands were equally precise (again, when the variable error was considered). In non musicians, pseudoneglect is typically stronger in haptic bisection with the left than with the right hand (Bradshaw et al., 1983), an effect that has been interpreted as reflecting further activation of the right hemisphere associated to the motor activation of the controlateral hand, this resulting into an increased leftward bias. Accordingly, we would have expected a stronger rightward bias with the right hand, ipsilateral to the bias. However, it might also be the case that the left hand may be always the more biased, regardless the (left vs. right) direction of the initial bias. Although this aspect cannot be clarified by our data and it is rather tangential to the scope of this work, it certainly deserves further investigation.

In conclusion, although previous studies have shown that sound frequency is represented in a spatial format and that the “music mental line” can affect bimanual motor responses (SMARC and SPARC effect) (Rusconi et al., 2006; Lidji et al., 2007; Nishimura and Yokosawa, 2009; Cho et al., 2012), our study provides the first evidence that pitch height influences the allocation of spatial attention crossmodally in tactile and visual peripersonal space in musicians.

## Conflict of interest statement

The authors declare that the research was conducted in the absence of any commercial or financial relationships that could be construed as a potential conflict of interest.
